# Severe ocular involvement in hereditary gelsolin amyloidosis

**DOI:** 10.1097/j.pbj.0000000000000146

**Published:** 2021-10-11

**Authors:** Nisa Filipa Pinho da Silva, João Nuno Melo Beirão

**Affiliations:** a Ophthalmology Department, Centro Hospitalar e Universitário do Porto, Porto, Portugal; b Instituto de Ciências Biomédicas Abel Salazar, University of Porto

**Keywords:** Hereditary gelsolin amyloidosis, Meretoja syndrome, famylial amyloidosis finish-type, corneal lattice amyloidosis

## Abstract

Hereditary gelsolin amyloidosis is a rare subtype of hereditary systemic amyloidosis. An old male presented with the characteristic triad of symptoms, including bilateral facial palsy, cutis laxa, and corneal lattice amyloidosis. The diagnosis was confirmed by the detection of the mutation in gelsolin gene located on chromosome 9. Although the presenting symptoms were highly suggestive of the disease, reports of severe ocular involvement are scarce in the literature.

To the editor

Amyloid fibrils are misfolded protein homopolymers that adopt a cross-β conformation. Amyloid is one of the most stable protein conformations and its deposition in organs or tissues leads to a range of diseases known as amyloidosis.^1^ Hereditary systemic amyloidosis is a group of autosomal dominant, late-onset disorders caused by mutations in the genes of plasma proteins. Among the diverse types of hereditary amyloidosis, transthyretin-related (ATTR) amyloidosis is the most common, resulting from mutations in the transthyretin gene located on chromosome 18. However, apolipoprotein A-1, gelsolin, fibrinogen A α, and lysozyme genes may also be mutated in hereditary amyloidosis.^2^

A 74-year-old male presented to the ophthalmology outpatient clinic with complaints of progressive bilateral visual loss and dry eye symptoms for several years. He had a recent diagnosis of bilateral facial palsy and cutis laxa in a neurologic evaluation. There was a family history of hereditary gelsolin amyloidosis. Genetic testing for the familial disease was conducted and confirmed the presence of the gelsolin gene mutation located on chromosome 9q33 (c.640G>A). The ophthalmic evaluation revealed a visual acuity of hand motion in the right eye and finger counting in the left eye. He had an ectropion, conjunctival injection, and mucous discharge in both eyes (Fig. 1). A sensorial right exotropia was additionally noted. In slit-lamp examination, there was significant corneal neovascularization and lipid keratopathy involving the visual axis of the right eye (Fig. 2A). In the left eye, 360-degree peripheral corneal neovascularization and central haze were visualized (Fig. 2B). A slit-lamp magnified view of the left eye showed corneal lattice amyloidosis in the mid corneal stroma and superficial stromal scarring (Fig. 2C). Severe dry eye disease was evident by bilateral punctate corneal erosions subsequently treated with artificial tears.

**Figure 1:**
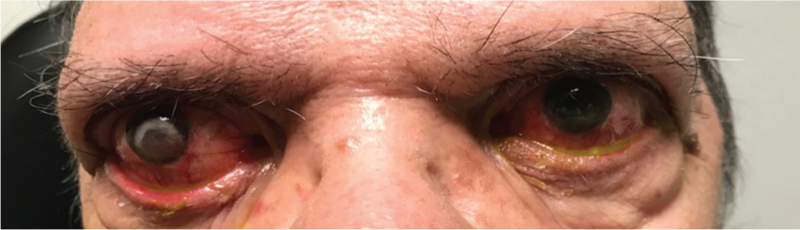
Frontal view of the patient showing ectropion (more pronounced in the right eye), conjunctival hyperemia and eyelids secretions with bilateral affection, and right exotropia. Cutis laxa was evident in the lower eyelids.

**Figure 2:**
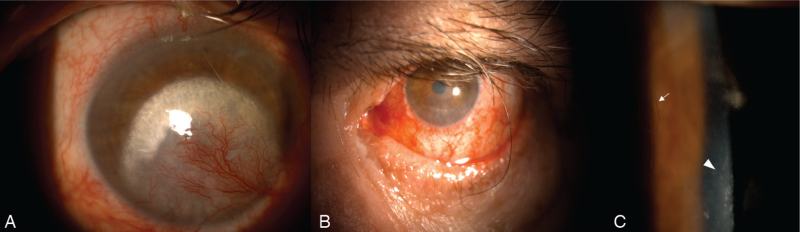
Slit-lamp photograph of the right (A) and left (B and C) eyes. (A) Corneal neovascularization and lipid keratopathy involving almost the entire cornea. (B) Peripheral corneal neovascularization affecting transparency in the periphery (also known as corneal pannus). An interface between peripheral neovascularized and central non-neovascularized cornea was evident. (C) Lattice lines of amyloid deposits (arrow) in the mid stroma and superficial scarring (arrowhead) in the magnified view of the cornea in (B).

Similar to ATTR amyloidosis, hereditary gelsolin (AGel) amyloidosis is a subtype of hereditary systemic amyloidosis with autosomal dominant inheritance, but this condition was described later (in 1969) by the Finnish ophthalmologist Jouko Meretoja.^3^ This doctor was from Finland, the country with the highest prevalence of the disease. Also, ATTR amyloidosis, described by the Portuguese Corino de Andrade in 1952,^4^ is considered to be endemic to Portugal. AGel amyloidosis is also known as Meretoja’ Syndrome or familial amyloidosis Finish-type, while ATTR amyloidosis is occasionally referred to as Corino de Andrade's disease or Portuguese-type amyloidosis. Noteworthy, few cases of AGel amyloidosis have been reported in Portugal.^5^ This type of hereditary amyloidosis is a very rare disease affecting fewer patients than ATTR amyloidosis worldwide. The mutation is located in the gelsolin gene on chromosome 9 at q33.2 locus with guanine being replaced with either adenine (c.640G>A) or, less frequently, thymidine (c.640G>T), and coding aspartic acid or tyrosine, respectively, instead of asparagine.^6^ The penetrance achieves 100% for the most frequent variant. The deposition of gelsolin-based amyloid fibrils and pre-amyloid oligomers in multiple organs and tissues is responsible for the clinical manifestations. The typical diagnostic triad includes neurological (paresthesia and bilateral facial palsy), dermatological (drooping eyelids and cutis laxa), and ophthalmological (dry eye disease and corneal lattice amyloidosis, previously incorrectly known as lattice corneal dystrophy type II) manifestations. The patients usually develop symptoms in the fourth and fifth decades. Rarely, cardiovascular disorders, including cardiac arrhythmias, and renal failure are present.^5,7^ In contrast to ATTR amyloidosis, the life expectancy does not seem to be decreased in patients with AGel amyloidosis.^8,9^ For unknown reasons, the severity of the disease varies remarkably between patients, including those who are relatives. There is no cure for the disease and, hence, treatment is aimed at relieving symptoms.

In this case of AGel amyloidosis, the diagnosis was straightforward due to the presence of the typical triad of symptoms and family history. However, the particular interest of this case lies in the severity of the ocular involvement with no similar cases reported in the literature.
